# Impact of implementing a critical care team in an open general ICU

**DOI:** 10.1186/cc9891

**Published:** 2011-03-11

**Authors:** S Kim, IH Kim, S Han, SS Ki, GR Chon

**Affiliations:** 1 Konkuk University Chungju Hospital, Chungju, South Korea

## Introduction

High-intensity ICU physician staffing is associated with reduced ICU mortality [1]. We formed a critical care team (CCT) that consisted of five teaching staff interested in critical care management. The CCT had been activated by each member of the team if needed and had provided rapid medical services including consultation. We evaluated the impact of implementing the CCT on open general ICU patient outcomes.

## Methods

We performed a prospective observational study in an open general ICU between March 2009 and February 2010 according to CCT. We compared demographic data, ICU mortality rates, length of ICU stay, APACHE II scores, Sequential Organ Failure Assessment (SOFA) scores, patients who received mechanical ventilation, and success rates of weaning in CCT with those in non-CCT.

## Results

We analyzed 857 patients' data (161 cases in CCT vs. 696 cases in non-CCT), excluding readmission cases. Patients who received CCT management were more severe than those who received non-CCT management significantly (APACHE II 21.4 vs. 17.7; SOFA 5.8 vs. 4.9). Although there were more patients on applied mechanical ventilation (46% vs. 23.6%) in CCT than those in non-CCT and a higher success rate of weaning (60.8% vs. 43.9%) in CCT than those in non-CCT, there was no significant difference of unadjusted ICU mortality rates in both groups (14.3% in CCT vs. 12.2% in non-CCT). Using a multivariate logistic regression model, the ICU mortality rate was associated with non-CCT, APACHE II scores, SOFA scores, and applied mechanical ventilation (Table 1).

**Table 1:** Multivariate logistic regression analysis of factors affecting ICU mortality

	*P *value
Male	0.864
Age	0.237
LOS, ICU	0.281
CCT	0.049
APACHE II	0.012
SOFA	0.004
Mechanical ventilation	< 0.001

## Conclusions

Although the CCT was not a full-time coverage team in the open general ICU, the CCT model was associated with reduced ICU mortality, especially in patients who received mechanical ventilation.
